# Treatment effect of intravenous high-dose selenium in sepsis phenotypes: a retrospective analysis of a large multicenter randomized controlled trial

**DOI:** 10.1186/s40560-025-00790-2

**Published:** 2025-04-14

**Authors:** David I. Radke, Holger Bogatsch, Christoph Engel, Frank Bloos, Patrick Meybohm, Michael Bauer, Anna Lulu Homayr, Christian Stoppe, Gunnar Elke, Matthias Lindner, the SepNet Critical Care Trials Group, Axel Nierhaus, Josef Briegel, Ulrich Jaschinski, Onnen Mörer, Andreas Weyland, Matthias Gründling, Stefan Kluge, Stefan Utzolino, Sebastian Stehr, Maximilian Ragaller, Frank Brunkhorst, Herwig Gerlach

**Affiliations:** 1 Department of Anaesthesiology and Intensive Care Medicine, University Medical Center Schleswig-Holstein, Campus Kiel, Arnold-Heller-Str. 3, 24105 Kiel, Germany; 2 Clinical Trial Centre Leipzig, Leipzig University, Leipzig, Germany; 3 Institute for Medical Informatics, Statistics and Epidemiology, Leipzig University, Leipzig, Germany; 4 Department of Anesthesiology and Intensive Care Medicine, Jena University Hospital, Jena, Germany; 5 Department of Anaesthesiology, Intensive Care, Emergency and Pain Medicine, University Hospital Würzburg, Würzburg, Germany; 6 Department of Cardiac Anesthesiology and Intensive Care Medicine, Charité Berlin, Berlin, Germany

**Keywords:** Sepsis, Septic shock, Sodium selenite, Sepsis phenotypes, Individualized medicine, Oxidative stress, Pharmaconutrition, Micronutrient

## Abstract

**Background:**

Treatment effect of high-dose intravenous selenium remains controversial in patients with sepsis or septic shock. Here, we reanalyzed data from the randomized placebo-controlled trial of Sodium Selenite and Procalcitonin Guided Antimicrobial Therapy in Severe Sepsis (SISPCT) to reveal possible treatment differences according to established sepsis phenotypes.

**Methods:**

In this secondary data analysis of the SISPCT trial all 1089 patients of the original study were included. Patients were assigned to one of the four phenotypes by comparing patient variables with the Sepsis Endotyping in Emergency Care (SENECA) validation cohort. Survival analyses were performed using Kaplan–Meier and log-rank tests.

**Results:**

No robust effect of selenium on mortality and other outcome parameters could be determined in any sepsis phenotype. Phenotype frequencies were markedly different in our study cohort compared to previous reports (*α*: 2.2%, *β*: 6.3%, *γ*: 68.0%, *δ*: 23.4%). Differences in mortality between the respective phenotypes were not significant overall; however, 28-day mortality showed a lower mortality for the *α*- (20.8%) and *β*-phenotype (20.3%), followed by the *γ*- (27.1%), and *δ*-phenotype (28.5%).

**Conclusions:**

Application of the four sepsis phenotypes to the SISPCT study cohort showed discrete but non-significant mortality differences within 28 days. However, beneficial treatment effects of high-dose intravenous selenium were still not detectable after categorizing the SISPCT study cohort according to four phenotype criteria.

**Supplementary Information:**

The online version contains supplementary material available at 10.1186/s40560-025-00790-2.

## Background

Sepsis and septic shock are frequent syndromes in critically ill patients with high mortality worldwide [1]. Inflammation and following oxidative stress is considered a crucial part of the pathophysiology of the host’s misguided immune-response, culminating in inflammation-triggered organ dysfunction [2]. In this context, selenium as an antioxidative micronutrient treatment strategy may restore glutathione peroxidase activity in sepsis [3, 4]. Glutathione peroxidases catalyze the reduction of hydrogen peroxide and lipid hydroperoxides, thereby preventing the accumulation of reactive oxygen species leading to diminished oxidative stress. In addition, selenium-dependent thioredoxin reductases keep thioredoxin in its reduced form. This is essential for DNA synthesis, protein repair, and antioxidant defense. A selenium deficiency compromises these pathways, increasing susceptibility to inflammatory damage [5]. Clinical data demonstrated that low plasma levels of selenium were adversely associated with patients’ outcome and sepsis mortality [6]. Except for one large randomized controlled trial (RCT) [7], recent RCTs in patients with sepsis or septic shock [8, 9], failed to show a clinical benefit of this pathophysiologically plausible pharmacological approach [2, 10]. In the Sodium Selenite and Procalcitonin Guided Antimicrobial Therapy in Severe Sepsis (SISPCT) trial, 28-day mortality was non-significantly different with 28.3% in the sodium selenite as opposed to 25.5% in the placebo group [8].

One possible explanation is that an undifferentiated treatment approach for sepsis in general may be inadequate in the presence of different inter- and intraindividual states of inflammation during this syndrome of different underlying diseases [11]. To face a more individualized approach, Seymour and colleagues defined and validated the four phenotypes of sepsis α, β, γ, and δ, where in-hospital mortality was shown to increase from α to δ from 2 to 32% [12]. Remarkably, conventional classification regimes such as severity of illness scores or focus of infection could not fully capture these phenotypes, indicating this method as a novel subgrouping approach [12].

According to results of a former RCT [7], we hypothesized that the antioxidative selenium treatment effect may rather depend on the severity of sepsis and the level of inflammation and hence on the individual sepsis phenotypes as opposed to the SEPSIS-1 criteria with a more pronounced responsiveness of the γ- and δ-phenotypes to selenium.

## Methods

### Study design and patients

This is a *post-hoc* analysis of the randomized placebo-controlled trial of Sodium Selenite and Procalcitonin Guided Antimicrobial Therapy in Severe Sepsis (SISPCT) trial, performed across 33 German multidisciplinary intensive care units (ICUs) from November 2009 until February 2013 [8]. SISPCT enrolled adults ≥ 18 years of age presenting with severe sepsis or septic shock according to the SEPSIS-1 definition with onset ≤ 24 h. Details of the SISPCT study design, data collection and management were described previously [8]. For the purpose of the present analysis, patients were further classified according to the phenotype criteria established by Seymour and coworkers [12]. We have chosen Seymour’s phenotyping strategy as we understand it as comprehensive, without limitation to single sepsis entities and, therefore, resembling the SISPCT trial, which also included patients with sepsis irrespective of the first organ manifestation.

### Statistical methods

Variables collected in the SISPCT trial were compared to the data obtained from the Sepsis Endotyping in Emergency Care (SENECA) validation cohort according to Seymour et al. [12]. The SENECA validation cohort is a retrospective data cohort containing 43,086 encountering patients fulfilling Sepsis-3 criteria in 12 North American hospitals in 2013 and 2014. In-hospital mortality was recorded as primary outcome [12]. Patients were phenotyped by calculating the Euclidean distance from each individual to the centroid of the respective phenotype as described in the supplementary material by Seymour and colleagues [12]. Missing data at baseline were imputed with data from day 0 or, if day 0 was not available, from day 1 (Table S1 in Supplement). Nine clinical variables for phenotyping were missing in the SISPCT data set. This was comparable to four study data sets used by Seymour et al. to verify their phenotyping method [12]. As Seymour et al. refer to the new SEPSIS-3-definition, we have added the SOFA-score for clustering to reduce the limitations of SEPSIS-1. SOFA scores at baseline were available for *n* = 1021 patients, imputation was available for *n* = 42 patients. 26 patients remained without SOFA score. In case of multiple values, the respective minimum or maximum was considered according to the supplementary material by Seymour and colleagues. Categorical data were reported as absolute or relative frequencies. Continuous data were presented by median and interquartile range. Due to the descriptive nature of this *post-hoc*-analysis and the small number of patients in phenotype α and β, significance tests were largely omitted. Kaplan–Meier and log-rank tests were used for survival analyses. Statistical analyses were performed with SPSS version 29.0.0.0 (IBM SPSS Statistics).

## Results

### Patient characteristics according to phenotypes

**Fig. 1:**
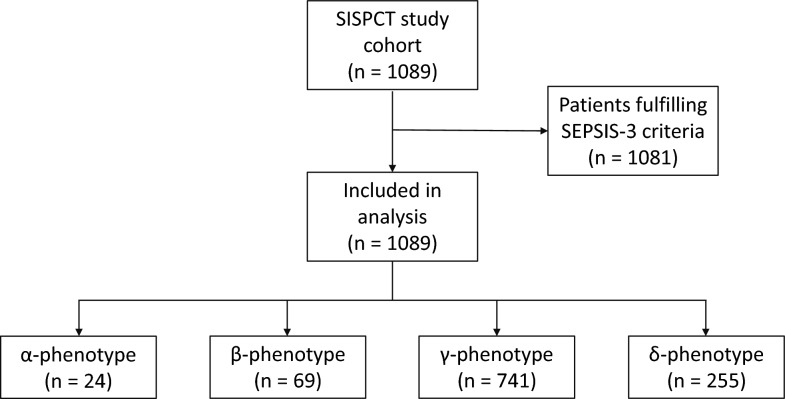
Flowchart of study cohort

The entire SISPCT study population consisting of 1089 patients with either severe sepsis (*n* = 142; 13%) or septic shock (*n* = 947; 87%) according to Sepsis-1 definition was considered for analysis. Of these patients 1081 fulfilled criteria for the SEPSIS-3 definition. In detail, 467 (43.2%) and 614 (56.8%) were categorized in sepsis and septic shock according to the SEPSIS-3 definition, respectively. 24 patients were categorized according to the α-phenotype (2.2%), 69 to the β-phenotype (6.3%), 741 met the criteria for the γ-phenotype (68.0%), and 255 individuals for the δ-phenotype (23.4%) (Fig. 1).

Patient characteristics of the total study population and according to sepsis phenotypes upon study enrollment are summarized in Table 1.

**Table 1:** Baseline characteristics and phenotypes

Characteristics study cohort	Total SEPSIS-1	Phenotype			
		α	β	γ	δ
No. of patients (%)	1089	24 (2.2)	69 (6.3)	741 (68.0)	255 (23.4)
Age, mean, median [IQR], year	68 [57–75]	67 [57–76]	75 [69–80]	67 [56–74]	70 [59–77]
Sex, No. (%)					
Male	691 (63.5)	19 (79.2)	36 (52.2)	487 (65.7)	149 (58.4)
Female	398 (36.5)	5 (20.8)	33 (47.8)	254 (34.3)	106 (41.6)
Septic shock (SEPSIS-3), No. (%)	614/1081 (56.8)	13/24 (54.2)	35/68 (51.5)	415/739 (56.2)	151/250 (60.4)
Charlson Comorbidity Index, median [IQR]	2 [1–4]	2.5 [0.3–3.8]	3 [2–4]	2 [1–4]	2 [1–4]
Type of admission, No. (%)					
Emergency surgery	494 (45.4)	11 (45.8)	40 (58.0)	304 (41.0)	139 (54.5)
Emergency (non-surgical)	473 (43.4)	10 (41.7)	21 (30.4)	345 (46.6)	97 (38.0)
Elective surgery	122 (11.2)	3 (12.5)	8 (11.6)	92 (12.4)	19 (7.5)
Reached maximum within 24 h, median [IQR]					
SIRS criteria	4 [3, 4]	3 [2, 3]	3 [3, 4]	4 [3, 4]	4 [3, 4]
SOFA Score^a^	10 [8–12]	10 [9–12]	9 [7–10]	10 [8–12]	9 [7–11]
Inflammation					
CRP, median [IQR], mg/L^b^	190 [120–284]	141 [80–239]	231 [122–300]	197 [122–290]	178 [108–266]
PCT, median [IQR], ng/mL	7.4 [1.6–26.8]	1.4 [0.3–8.1]	5.7 [1.4–25.7]	8.0 [1.7–29.8]	6.1 [1.7–23.5]
Temperature, median [IQR], °C^b^	38.0 [37.2–38.7]	37.9 [36.8–38.7]	37.4 [36.8–38.4]	38.1 [37.3–38.8]	37.6 [37.0–38.4]
White blood cell count, median [IQR], × 10^9^/L^b^	15.8 [10.3–22.7]	15.8 [12.7–24.2]	14.9 [9.8–23.1]	16.0 [10.4–23.2]	15.3 [10.1–21.0]
Pulmonary					
PaO_2_, median [IQR], mmHg^b^	78 [68–99]	98 [84–101]	99 [95–105]	71 [63–80]	123 [107–154]
PaO_2_/FiO_2_-ratio, median [IQR], mmHg^b^	158 [107–230]	199 [128–255]	228 [180–300]	138 [93–184]	253 [170–331]
Respiratory rate, median [IQR], breaths/min^b^	24 [20–30]	22 [18–30]	21 [17–24]	25 [20–31]	22 [17–29]
Cardiovascular or Hemodynamic					
Bicarbonate, median [IQR] mmol/L^b^	20.9 [17.9–24.0]	21.5 [18.4–24.5]	20.8 [17.7–24.1]	21.0 [18.0–24.3]	20.2 [17.3–22.9]
Heart rate, median [IQR], beats/min^b^	120 [104–136]	71 [62–84]	97 [85–102]	120 [107–137]	125 [110–140]
Serum lactate, median [IQR], mmol/L^b^	2.7 [1.6–4.7]	2.2 [1.4–2.9]	2.3 [1.5–4.0]	2.7 [1.6–4.7]	3.0 [1.7–4.8]
Systolic blood pressure, median [IQR], mmHg^b^	83 [74–94]	91 [82–110]	80 [72–93]	84 [75–93]	80 [70–94]
Renal					
Creatinine, median [IQR], µmol/L^b^	133 [88–212]	124 [77–225]	168 [106–279]	133 [88–212]	124 [80–211]
Blood urea, median [IQR], mmol/L^b^	9.8 [6.1–16.0]	8.5 [3.9–15.9]	11.3 [7.9–21.1]	9.8 [6.3–15.6]	9.2 [5.5–16.8]
Hepatic					
Bilirubin, median [IQR], µmol/L^b^	15.4 [8.6–26.0]	17.6 [9.0–34.8]	16.0 [9.4–25.7]	15.4 [8.6–27.4]	13.7 [8.6–22.2]
Hematologic					
Hemoglobin, median [IQR], mmol/L^b^	5.8 [5.2–6.9]	6.1 [5.4–7.2]	5.8 [5.2–6.8]	5.8 [5.2–6.8]	5.9 [5.0–7.0]
Platelets, median [IQR], × 10^9^/L^b^	199 [132–286]	224 [140–320]	181 [145–261]	195 [125–289]	208 [143–287]
Other					
Albumin, median [IQR], g/L^b^	20 [15–25]	29 [21–34]	17 [14–21]	20 [16–25]	19 [15–24]
Glucose, median [IQR], mmol/L^b^	8.8 [7.4–10.8]	8.3 [7.0–10.0]	8.7 [6.9–10.4]	8.9 [7.5–10.9]	8.5 [7.2–10.4]
Sodium, median [IQR], mmol/L^b^	140 [137–144]	143 [137–145]	139 [136–142]	141 [137–144]	140 [135–143]
GCS, median [IQR]^b^	15 [10–15]	14 [4–15]	15 [14, 15]	15 [10–15]	15 [11–15]
Mechanical ventilation, median days [IQR]^c^	6 [1–15]	11 [2–24]	2 [1–6]	7 [2–17]	4 [1–10]
Renal replacement therapy, median days [IQR]^c^	0 [0–1]	0 [0–1]	0 [0–1]	0 [0–2]	0 [0–1]
Vasopressor use, median days [IQR]^c^	3 [1–9]	9 [3–16]	2 [1–5]	4 [1–9]	3 [1–7]
Antibiotic use, median days [IQR]^c^	7 [3–15]	10 [6–20]	6 [3–12]	8 [4–16]	6 [3–12]
In-hospital mortality, *N* (%) [95% CI]	342/1027 (33.3) [30.5–36.2]	7/24 (29.2) [14.9–49.2]	19/65 (29.2) [19.6–41.2]	238/696 (34.2) [30.8–37.8]	78/242 (32.2) [26.7–38.4]
28-day mortality, *N* (%) [95% CI]	289/1076 (26.9) [24.3–29.6]	5/24 (20.8) [9.2–40.5]	14/69 (20.3) [12.5–31.2]	198/790 (27.1) [22.2–28.2]	72/253 (28.5) [23.3–34.3]
90-day mortality, *N* (%) [95% CI]	399/1045 (38.2) [35.3–41.2]	9/24 (37.5) [21.2–57.3]	27/68 (39.7) [28.9–51.6]	271/706 (38.4) [34.9–42.0]	92/247 (37.2) [31.5–43.4]

IQR: interquartile range; SIRS: systemic inflammatory response syndrome; SOFA: sequential organ failure assessment; CRP: C-reactive protein; PCT: procalcitonin

^a^Missing subscores imputed by values at day 0 or day 1

^b^Missing values imputed by values at day 0 or day 1

^c^Until day 90 (ICU)

The β-phenotype in the cohort contained the oldest individuals. The entire SISPCT study population showed a pronounced sex imbalance in favor of male individuals (63.5% vs. 36.5%). This distribution was even more pronounced in the α-phenotype (79.2% male vs. 20.8% female), whereas this was rather comparable in the β-phenotype group (52.2% male vs. 47.8% female). Highest CRP levels were seen in the β- (231 mg/L, IQR 122–300) followed by the γ-phenotype (197 mg/L, IQR 122–290). PCT values were strongly elevated in the γ-phenotype (8.0 ng/mL, IQR 1.7–29.8), concomitant with highest body temperature (38.1 °C, IQR 37.3–38.8) and white blood cell count (16.0 × 10^9^/L, IQR 10.4–23.2). The PaO_2_/FiO_2_-ratio was lowest in the γ-phenotype (138 mmHg, IQR 93–184). The γ- and δ-phenotype showed a higher median heart rate than the other two phenotypes. Highest creatinine and urea blood levels were seen in the β-phenotype. The lowest albumin levels appeared also in the β-phenotype group, followed by the δ-phenotype (17 g/L, IQR 14–21 and 19 g/L, IQR 15–24, respectively), whereas the α-phenotype showed the highest value of serum albumin (29 g/L, IQR 21–34). Bilirubin was lowest in the δ-phenotype.

### Selenium effect on outcome

**Fig. 2:**
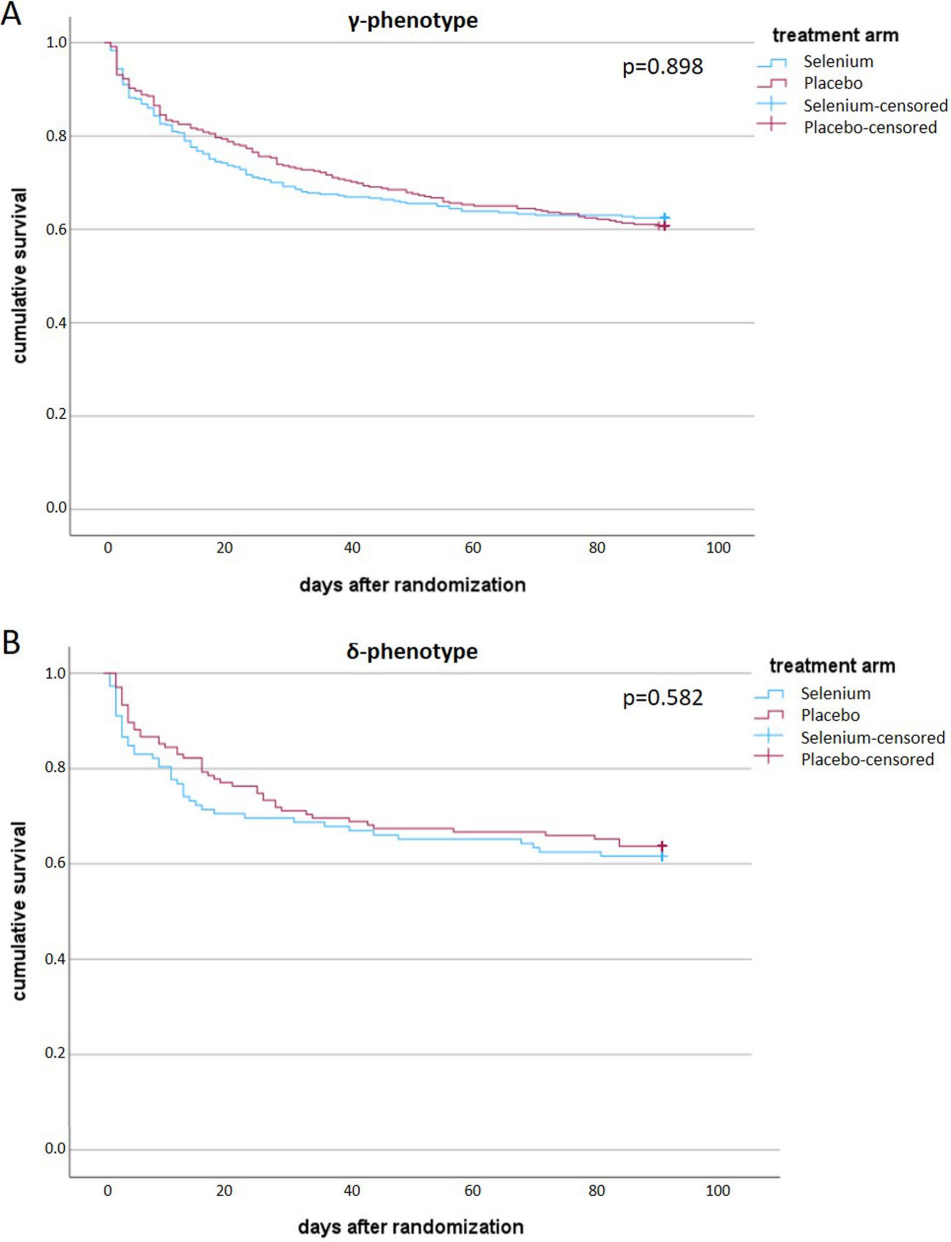
Survival functions of study population treated with selenium or placebo clustered in phenotypes γ and δ after 90 days. **A** shows cumulative survival of γ-phenotype, **B** shows cumulative survival of δ-phenotype. α- and β-phenotype not shown

In the α-phenotype group, selenium treatment was associated with reduced in-hospital, 28-day, and 90-day mortality and higher mortality rates in β-phenotype patients (Table 2). For the γ- and δ-phenotype no significant differences were observed comparing placebo- and selenium-treated patients (γ: *p* = 0.898; δ: *p* = 0.582). No obvious differences in hospital- or ICU-length of stay were observed. Numbers of individuals in the respective phenotype were unevenly distributed (Table 2 and Fig. 2).

**Table 2:** Selenium effect on outcome according to phenotypes

Characteristics study cohort	Phenotype							
	α		β		γ		δ	
	Selenium (*n* = 10)	Placebo (*n* = 14)	Selenium (*n* = 39)	Placebo (*n* = 30)	Selenium (*n* = 378)	Placebo (*n* = 363)	Selenium (*n* = 116)	Placebo (*n* = 139)
Mechanical ventilation, median days [IQR]^a^	7 [2–27]	15 [2–23]	2 [1–6]	2 [1–6]	6 [2–15]	7 [2–19]	3 [1–9]	4 [1–12]
Renal replacement therapy, median days [IQR]^a^	0 [0–0]	0 [0–4]	0 [0–2]	0 [0–1]	0 [0–2]	0 [0–2]	0 [0–1]	0 [0–1]
Vasopressor use, median days, [IQR]^a^	4 [1–17]	11.5 [3–17]	2 [1–5]	2 [1–6]	3 [1–9]	4 [1–10]	2 [1–6]	3 [1–7]
Antibiotic use, median days [IQR]^a^	8 [5–19]	11 [8–21]	5 [2–10]	8 [4–13]	7 [4–14]	8 [4–17]	6 [2–10]	7 [3–14]
In-hospital mortality, No. (%) [95% CI]	1/10 (10) [1.8–40.4]	6/14 (42.9) [21.4–67.4]	14/39 (35.9) [22.7–51.6]	5/26 (19.2) [8.5–37.9]	123/349 (35.2) [30.4–40.4]	115/347 (33.1) [28.4–38.3]	35/111 (31.5) [23.6–40.7]	43/131 (32.8) [25.4–41.3]
28-day mortality, No. (%) [95% CI]	1/10 (10) [1.8–40.4]	4/14 (28.6) [11.7–54.7]	10/39 (25.6) [14.6–41.1]	4/30 (13.3) [5.3–29.7]	107/373 (28.7) [24.3–33.5]	91/357 (25.5) [21.3–30.3]	34/116 (29.3) [21.8–38.2]	38/137 (27.7) [20.9–35.8]
90-day mortality, No. (%) [95% CI]	1/10 (10.0) [1.8–40.4]	8/14 (57.1) [32.6–78.6]	20/38 (52.6) [37.3–67.5]	7/30 (23.3) [11.8–40.9]	134/357 (37.5) [32.7–42.7]	137/349 (39.3) [34.3–44.5]	43/112 (38.4) [29.9–47.6]	49/135 (36.3) [28.7–44.7]
LOS ICU, median days [IQR]	16 [8–34]	19 [12–25]	8 [4–15]	11 [5–18]	12 [6–25]	13 [6–26]	9 [4–14]	11 [6–20]
LOS hospital, median days [IQR]	36 [19–45]	34 [27–50]	27 [17–39]	33 [23–55]	27 [17–42]	29 [17–49]	24 [15–42]	28 [16–47]

IQR: interquartile range; 95% CI: 95% confidence interval

^a^Until day 90 (ICU)

Subsequently, further outcome parameters of the study population clustered into phenotypes in response to selenium treatment were analyzed. Median duration of mechanical ventilation was lowest in the selenium treated α-phenotype group, as well as median time of vasopressor and antibiotic use. Median time of antibiotic use was lowest in the selenium treated β-phenotype. No selenium effect on the respective outcome parameters were detectable in the γ- and δ-phenotype groups (Table 2).

### Outcome according to phenotypes

**Fig. 3:**
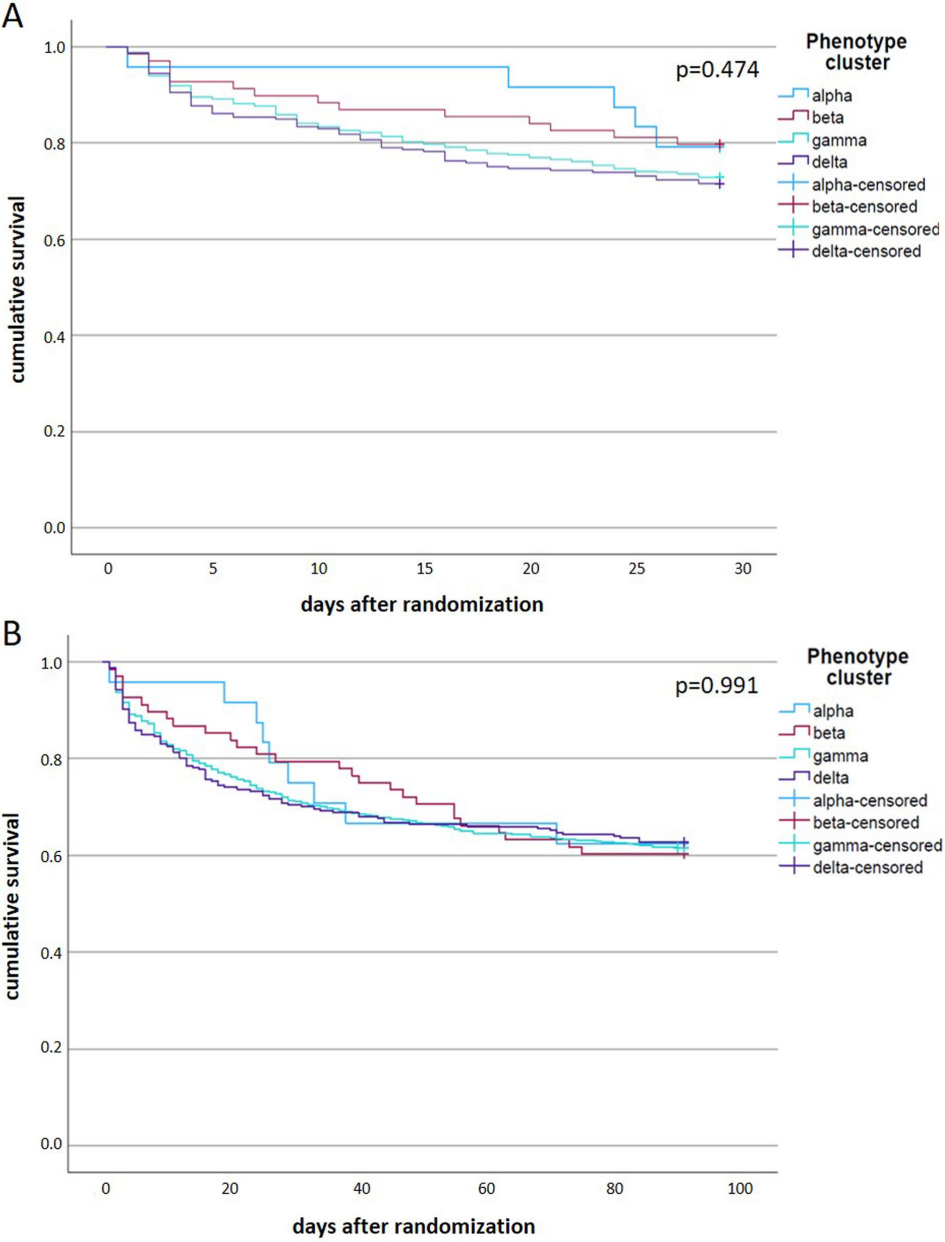
Survival functions according to phenotypes. **A** shows cumulative survival after 28 days, **B** shows cumulative survival after 90 days

28-day all-cause mortality in the entire study cohort was 26.9%. In the following step, we examined the 28-day mortality after applying the phenotypes to the study population. The α- and β-phenotype showed a similar mortality rate of 20.8% and 20.3%, respectively. The γ-phenotype had a 28-day mortality of 27.1% and the δ-phenotype showed the highest mortality (28.5%). Differences were not significant. 28- and 90-day mortality rates and further outcome parameters are listed in Table 1. Kaplan–Meier plots for 28- (*p* = 0.474) and 90-day (*p* = 0.991) mortality are shown in Fig. 3.

## Discussion

In recent years, results from large multicenter RCTs in patients with sepsis therapies including nutrition therapy and high-dose supplementation of micronutrients have essentially been negative or neutral and were, therefore, unable to confirm previous positive findings for (patho-) physiologically plausible therapies [13]. Reasons for this divergence were attributed particularly to the study design itself (e.g., choice of timing and dosing of a drug, synergistic effects seldom investigated, choice of unsuitable outcomes), inappropriate individualization of the intervention and phenotyping of the syndrome sepsis based solely on the clinically established SEPSIS-1 criteria [14–16].

Precision medicine approaches, that target patients based on disease subtypes, have transformed treatment approaches for malignancies, asthma and other heterogeneous syndromes and offer equally great potential in critical illness. Innovative approaches even postulate that it is necessary to abandon classical models such as the grouping of different symptoms into syndromes [11]. We obtained analogous results of the missing selenium treatment effect in septic patients, also after applying the subgrouping strategy, as we must note that numbers of individuals in the α- and β-phenotype divided into selenium and placebo group were too small to draw a valid conclusion. These data are, therefore, more of a descriptive nature. However, differences seen in outcome parameters in the α- and β-phenotype should be reevaluated in future studies. Focusing on these two phenotypes in particular to generate a sufficient number of individuals could ultimately answer the question of whether selenium is indicated in any subtype of septic patient. At this point, however, we are still unable to make a recommendation for application of high-dose sodium selenite in septic patients. In agreement with this, a recent systematic review and meta-analysis (SRMA) of 24 trials could not demonstrate a beneficial outcome as a consequence of high-dose selenium application in ICU-patients [17]. On the contrary, doses of more than 1000 µg per day were associated with increased length of stay (LOS) on the ICU [17]. Another SRMA, including 34 studies with 4678 patients, showed a tendency towards reduction of all-cause mortality by antioxidative micronutrient supplementation, albeit the authors did not consider these results to be robust. ICU–LOS and duration of mechanical ventilation therapy were reduced significantly by antioxidant administration [18]. Of note, in this SRMA antioxidative substances were summarized (selenium, zinc, vitamin A, C and E), although selenium accounted for the largest proportion. In 2022, Gudivada and coworkers found in their SRMA further results suggestive of beneficial outcome (e.g., LOS, ventilator days, infections) of ICU patients, again investigating antioxidative cocktails and not selenium as monotherapy, however [19]. In addition, supplementation of high-dose selenium did not improve postoperative organ dysfunction or mortality in cardiac surgery patients, a patient group that is known to have a pronounced inflammatory reaction, as it was shown recently in a large international multicenter trial [20]. As it is hypothesized that reactive oxygen species are to some extent essential for a functioning immune response, antioxidant strategies may, therefore, deteriorate outcomes in late immunosuppressive states of sepsis [21]. Measurements of markers of oxidative stress in blood samples might help to predict the effect of selenium and other antioxidative supplements on outcome parameters. However, we are unable to provide information on oxidative stress levels as measurements were not carried out in the SISPCT trial.

Analyzing further patient characteristics and outcomes, we confirmed, in line with Seymour’s investigation, that patients attributed to the β-phenotype were oldest and had the highest serum levels of creatinine and urea, indicating a higher proportion of renal dysfunction. Furthermore, signs of inflammation such as PCT levels and body temperature were, like in the original work, most elevated in the γ-phenotype, except for plasma CRP levels which were higher in the β-phenotype group. In addition, inconsistent with Seymour’s work, we found that the lowest albumin levels were not found in the γ- but in the β-phenotype. Liver dysfunction, reflected by bilirubin levels, which were highest in Seymour’s δ-phenotype, were on the contrary lowest in the same phenotype of our cohort. Different inclusion criteria in the respective studies are likely to account for these variations, as, for example, liver cirrhosis was an exclusion criterion in the SISPCT-trial [8].

Seymour and colleagues observed an increase in in-hospital deaths, 28- and 365-day mortality from the α- to the δ-phenotype [12]. Interestingly, in the studies analyzed by this group the β- and γ-phenotype showed a comparable mortality rate, whereas patients attributed to the α- and to the δ-phenotype had a considerably lower or higher respective mortality. When looking at the absolute values for early mortality, it is striking that especially the α-phenotype showed a remarkably low mortality-rate varying between 5 and 16% in the original publication compared 20.8% in this study. On the other hand, both early and late mortality in the δ-phenotype group was evidently higher in the original study. In our study a more pronounced, but nonetheless insignificant, gradation of the mortality-rate values was observed at an even earlier timepoint, such as mortality until day 28. In contrast to the phenotype-defining work, we could not detect differences in long-term mortality between the four phenotypes. The mortality in the entire SISPCT trial was comparable to other clinical studies performed by the SepNet Critical Care Trials Group, e.g., the VISEP trial [22] or the MAXSEP trial [23]. However, epidemiological studies like the INSEP trial [24] showed even higher mortality rates which can be explained by different inclusion criteria, sepsis origin, and ultimately by the size of the study population.

In the present study, the four sepsis phenotypes were applied retrospectively to the SISPCT trial and different frequency distributions of the individual phenotype were observed. In our cohort the γ-phenotype was the most common, followed by the δ-phenotype. The α-phenotype was on the other hand the rarest. This indicates a higher proportion of more severely ill patients. Suitably, comparing the SOFA score for the two study cohorts, the SISPCT-cohort had higher values (mean 10 vs. 3.9 points). However, the substantially lower incidence of the β-phenotype in our study cohort (6.3% vs. 27%), representing the phenotype with the most elderly patients likely to suffer from renal dysfunction, was neither reflected by the mean age (68 years in SISPCT vs. 64 years in the Seymour study), nor by mean laboratory markers of kidney function (creatinine 133 µmol/L and blood urea 9.8 mmol/L in SISPCT vs. creatinine 124 µmol/L and blood urea 8.6 mmol/L in the Seymour study) in the respective study cohorts. Recently, the sepsis phenotypes were applied for COVID and non-COVID sepsis patients [25]. Interestingly, the authors also found a different frequency distribution of phenotypes compared to the original publication. This study cohort consisted, like the SISPCT cohort, exclusively of patients who had already been admitted to the ICU, whereas Seymour and coworkers investigated a more inhomogeneous cohort. When considering patients with bacterial pneumonia, the authors found a similar frequency distribution of phenotypes as we did. In line with this observation the largest group of the SISPCT-cohort had a pulmonary focus [8]. In a secondary analysis of the PROWESS trial a pulmonary focus of infection increased the proportion of the γ-phenotype, supporting our data [26]. Although in the original publication by Seymour site of infection could not describe the phenotypes sufficiently, it may have at least an impact on the respective phenotype’s abundance.

In addition to the limitations inherent in the study design of a retrospective analysis, we only examined one trial as a proof-of-concept study. Larger data sets are required for generalizability to gain further insights into potential different treatment effects according to phenotype categorization as opposed to the clinically established SEPSIS-1 standard. Although we used multiple imputation for missing data at baseline, bias cannot be ruled out. Furthermore, we only applied one phenotyping strategy to the SISPCT study cohort. Whether other already established phenotyping methods show differential selenium treatment effects, e.g., hypo- versus hyperinflammatory sepsis cannot be derived from our study population but should be addressed in further clinical trials. Strength of this study is that it analyses a large and well-described cohort from a randomized multicenter RCT, including 33 multidisciplinary ICUs. Data of patients’ characteristics were documented diligently until day 21 with high adherence to the study protocol. Finally, mortality rates were recorded up to both day 28 and day 90.

## Conclusion

Supplementation of high-dose selenium was not associated with mortality and other clinical outcomes measured when sepsis or septic shock were recategorized according to the four sepsis phenotypes. 28- and 90-day mortality were non-significantly different among the phenotypes.

## Supplementary Information

Additional file 1: Table S1. Variables of SENECA validation cohort included in analysis.

## Data Availability

The data sets used and/or analyzed during the present study are available from the corresponding author on reasonable request.

## Abbreviations

CI: Confidence interval
COVID: Coronavirus disease
CRP: C-reactive protein
FiO_2_: Fraction of inspired oxygen
GCS: Glasgow coma score
ICU: Intensive care unit
IQR: Interquartile range
LOS: Length of stay
PaO_2_: Partial pressure of oxygen
PCT: Procalcitonin
RCT: Randomized controlled trial
SIRS: Systemic inflammatory response syndrome
SISPCT: Trial of Sodium Selenite and Procalcitonin Guided Antimicrobial Therapy in Severe Sepsis
SOFA: Sequential organ failure assessment
SRMA: Systematic review and meta-analysis
