# Supplementary material for: Treatment effect of intravenous high-dose selenium in sepsis phenotypes: a retrospective analysis of a large multicenter randomized controlled trial
Source: J Intensive Care. 2025 Apr 14;13:21. doi: 10.1186/s40560-025-00790-2 (PMC11995462; doi:10.1186/s40560-025-00790-2)
Supplement: Supplementary file 1 — Additional file 1: Table S1. Variables of SENECA validation cohort included in analysis. [file 40560_2025_790_MOESM1_ESM.docx]

Additional file 1

Table S1. Variables of SENECA validation cohort included in analysis

| **Variable** | **SENECA validation cohort** | **SISPCT** |
| --- | --- | --- |
| **Age** | X | X |
| **Albumin** | X | X |
| **Alanine Aminotransferase** | X | Not available |
| **Aspartate Aminotransferase** | X | Not available |
| **Bands** | X | Not available |
| **Bicarbonate** | X | X |
| **Bilirubin** | X | X |
| **Blood urea nitrogen** | X | Urea |
| **Chloride** | X | X |
| **C-reactive protein** | X | X |
| **Creatinine** | X | X |
| **Elixhauser** | X | Not available |
| **Erythrocyte sedimentation rate** | X | Not available |
| **Glasgow coma score** | X | X |
| **Glucose** | X | X |
| **Heart rate** | X | X |
| **Hemoglobin** | X | X |
| **International normalized ratio** | X | Not available |
| **Lactate** | X | X |
| **Oxygen saturation** | X | Not available |
| **Partial pressure of oxygen** | X | X |
| **Platelets** | X | X |
| **Respiratory rate** | X | X |
| **Sex** | X | X |
| **Sodium** | X | Not available |
| **Systolic blood pressure** | X | X |
| **Temperature** | X | X |
| **Troponin** | X | Not available |
| **SOFA score** |  | X |
| **White blood cell count** | X | X |

We used eTable3 for variables and eTable12 for data from Seymour et al. 2019 for clustering (12).
